# Molecular characterization and genetic authentication assay for *Anopheles* ‘hemocyte-like’ cell lines 4a-3A and 4a-3B

**DOI:** 10.1186/s13071-022-05590-3

**Published:** 2022-12-13

**Authors:** Heather Eggleston, Kimani Njoya, Cameron E. Anderson, Inge Holm, Karin Eiglmeier, Jiangtao Liang, Igor V. Sharakhov, Kenneth D. Vernick, Michelle M. Riehle

**Affiliations:** 1 Department of Microbiology and Immunology, Medical College of Wisconsin, Milwaukee, WI USA; 2 Institut Pasteur, Université de Paris, CNRS UMR 2000, Unit of Insect Vector Genetics and Genomics, Department of Parasites and Insect Vectors, Paris, France; 3 Department of Entomology, Virginia Polytechnic Institute and State University, Blacksburg, VA 24061 USA

**Keywords:** *Anopheles gambiae*, *Anopheles coluzzii*, Hemocytes, Cell line authentication, Reproducibility

## Abstract

**Background:**

*Anopheles* cell lines are used in a variety of ways to better understand the major vectors of malaria in sub-Saharan Africa. Despite this, commonly used cell lines are not well characterized, and no tools are available for cell line identification and authentication.

**Methods:**

Utilizing whole genome sequencing, genomes of 4a-3A and 4a-3B ‘hemocyte-like’ cell lines were characterized for insertions and deletions (indels) and SNP variation. Genomic locations of distinguishing sequence variation and species origin of the cell lines were also examined. Unique indels were targeted to develop a PCR-based cell line authentication assay. Mitotic chromosomes were examined to survey the cytogenetic landscape for chromosome structure and copy number in the cell lines.

**Results:**

The 4a-3A and 4a-3B cell lines are female in origin and primarily of *Anopheles coluzzii* ancestry. Cytogenetic analysis indicates that the two cell lines are essentially diploid, with some relatively minor chromosome structural rearrangements. Whole-genome sequence was generated, and analysis indicated that SNPs and indels which differentiate the cell lines are clustered on the 2R chromosome in the regions of the 2Rb, 2Rc and 2Ru chromosomal inversions. A PCR-based authentication assay was developed to fingerprint three indels unique to each cell line. The assay distinguishes between 4a-3A and 4a-3B cells and also uniquely identifies two additional *An. coluzzii* cell lines tested, Ag55 and Sua4.0. The assay has the specificity to distinguish four cell lines and also has the sensitivity to detect cellular contamination within a sample of cultured cells.

**Conclusions:**

Genomic characterization of the 4a-3A and 4a-3B *Anopheles* cell lines was used to develop a simple diagnostic assay that can distinguish these cell lines within and across research laboratories. A cytogenetic survey indicated that the 4a-3A and Sua4.0 cell lines carry essentially normal diploid chromosomes, which makes them amenable to CRISPR/Cas9 genome editing. The presented simple authentication assay, coupled with screening for mycoplasma, will allow validation of the integrity of experimental resources and will promote greater experimental reproducibility of results.

**Graphical abstract:**

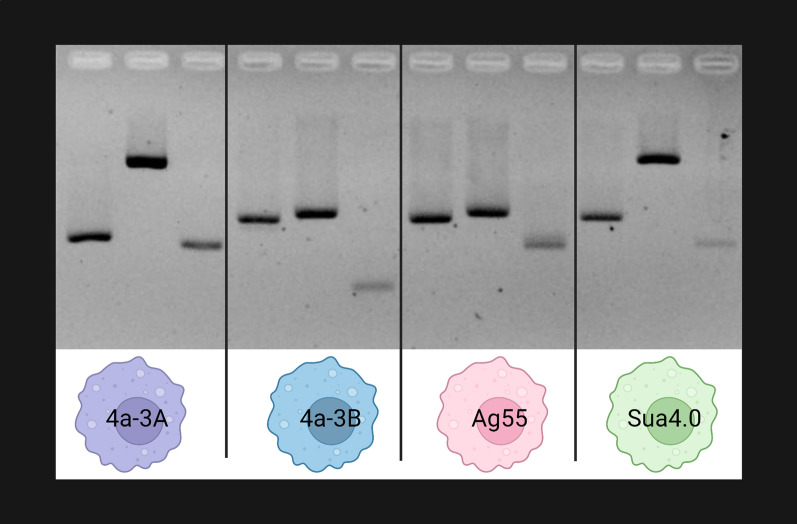

**Supplementary Information:**

The online version contains supplementary material available at 10.1186/s13071-022-05590-3.

## Background

*Anopheles* mosquitoes are major vectors of malaria parasites across sub-Saharan Africa. Vector control tools such as insecticide-treated bed nets and indoor residual spraying have long been the cornerstone of vector control strategies aimed at curbing malaria, arboviral and other vector-borne disease transmission. However, given increased insecticide resistance within mosquito populations, other control strategies are needed [1]. In vivo and in vitro experimental work on mosquito vectors continues to inform the best avenues for future control strategies. Laboratory colony mosquitoes and/or field-captured mosquitoes are often used in the in vivo work, and existing anopheline cell lines serve an essential role for in vitro studies. As with many biological systems, cell line models can allow for elegantly designed, carefully controlled in vitro experiments that can inform and target in vivo work and provide a means to screen a large number of candidate genes or proteins before pursuing work on a set of top candidates. Cell lines are also a valuable resource in the biochemical characterization of proteins.

There are over 500 insect cell lines originating from different tissues from species within Diptera, Lepidoptera and Hemiptera insect orders. Anopheline cell lines have been used to examine a wide variety of traits, including to determine if the Minos transposable element integrates in a transposase-dependent manner [2], to characterize the cellular response to Bin toxin, a candidate vector control tool [3, 4], to study hemocyte properties and prophenoloxidase (PPO) expression [5], for studies of phagocytosis and hemocyte gene expression [6], to characterize the effect of genetic variation enhancer activity [7], to characterize interaction of proteins with a role in malaria infection [8] and to perform STARR-seq and establish a genome-wide catalog of transcriptional enhancers [9]. Establishment of a male-specific anopheline cell line suitable for the study of processes unique to male mosquitoes was also recently reported [10]. The 4a-3B *Anopheles gambiae* cell line is the first continuous insect cell line used to study both humoral and cellular immune responses of *An. gambiae* mosquitoes, in particular prophenoloxidase biochemistry [5]. Numerous cell lines have also been established from the larvae of *An. stephensi*, a primary malaria vector in Southeast Asia [11, 12]. Cell-based screening platforms have been developed to identify new compounds that are lethal to mosquito cell lines but show little or no activity against other insects and are safe for human use [13]. *Aedes* cell lines are also frequently used to characterize the response to arboviral or other viral infections [14, 15]. Despite widespread use, cell lines are often poorly characterized, and tools are lacking to establish and confirm cell line identity within and across research laboratories.

Throughout the literature, many of the anopheline cell lines are described as *An. gambiae* in species origin, but most of these cell lines originated before the identification of *An. coluzzii*, the sister taxon to *An. gambiae* [16]. Furthermore, many of the cell lines originate from mosquito colonies that have possible hybrid species origins, including the G3 colony [17]. Given that many cell lines are morphologically similar, one challenge in the use of cell lines is their identification and authentication as well as the detection of cell line contamination. Often mosquito cell lines are exchanged between laboratories, and so any accidental mislabeling or contamination is simply passed along without means of verification or validation.

While tools to authenticate *Anopheles* cell lines were not available prior to the current work, similar tools are used and often required for publication of work on human cell lines. Cross contamination or misidentification of cells lines can lead to spurious results and a failure to replicate results. A panel of eight simple tandem repeats (STRs) and amelogenin for gender determination is adequate to distinguish and differentiate human cell lines, and protocols and commercial kits for authentication are widely available [18]. Papers have had to be retracted because of cell line contamination [19], and simple authentication assays have shown that results originally attributed to KU7 human cells should be attributed to HeLa cells because of contamination [20]. Recommendations are in place for authentication of human cell lines in an effort to avoid misidentification and spurious results [21]. STR assays have been developed to authenticate African green money cell lines and detect human contamination [22] and to distinguish intraspecific mouse cell lines [23]. Authentication of insect cell lines has been performed using denaturing gradient gel electrophoresis [24], and random amplified polymorphic DNA (RAPD) markers have been used to distinguish between 11 insect cell lines [25]. To date there are no methods to identify or distinguish among *Anopheles* cell lines. A simple molecular assay to distinguish cell lines would help to avoid spurious results and provide a means for standardization. Within a given laboratory there is a need to identify and distinguish cell lines from one another and across laboratories there is need to authenticate a given cell line.

Here, we perform whole genome sequencing of two *Anopheles* cell lines, 4a-3A and 4a-3B [5], and develop an authentication assay based on segregating indels to distinguish these cell lines from one another and also from two other commonly used *Anopheles* cell lines, Ag55 (Mos55) [26] and Sua4.0 [5]. Using whole genome sequences, we characterize the genomic distribution of variants distinguishing 4a-3A cells from 4a-3B cells as well as characterize the species origin and chromosomal inversion status of these cell lines. Cytogenetic examination indicated that the two cell lines carry an essentially normal diploid chromosome complement.

## Methods

### Sample collection and DNA extraction

#### Cell growth and DNA isolation

4a-3A, 4a-3B and Sua4.0 cells were grown at 27 °C without CO_2_ in Insect-XPRESS™ protein-free medium with l-glutamine (Lonza, Switzerland), supplemented with 10% heat-inactivated fetal bovine serum (FBS) in T-25 flasks, and cells were harvested by scraping upon reaching 80% confluency. Ag-55 cells were grown at 27 °C without CO_2_ in Leibovitz’s 15 media supplemented with 10% heat-inactivated FBS as previously described [3, 6] (Sigma, USA). Ag-55 cells were obtained from Dr. Michael Adang. 4a-3A, 4a-3B and Sua4.0 cells were obtained from the originator, Hans-Michael Müller, EMBL. Frozen stocks were generated upon cell receipt, and cells were then minimally passaged. Genome sequencing was performed on genomic DNA isolated at passage 21 (4a-3A cells) and passage 11 (4a-3B cells). Authentication assays were performed at low passage (< 20 passages) but there is no evidence that longer passage impacts assay performance.

Genomic DNA was isolated from harvested cells using DNAzol as previously described [27]. TruSeq Nano DNA sequencing libraries were prepared using standard protocols by the University of Minnesota Genomics Center and sequenced using NextSeq 150PE Illumina sequencing. The sequencing generated 73,994,878 reads for the 4a-3A sample (~ 37× coverage; assuming a 300 MB genome) and 71,851,100 reads for the 4a-3B sample (~ 36× coverage).

#### DNA sequencing and analysis

All raw FASTQ files have been deposited at the SRA public archive under accession number PRJNA842420. Following fastqc (v0.11.9) [28] quality checking of the 4a-3A and 4a-3B fastq files, Trimmomatic (v0.38) [29] was used to remove low-quality bases (SLIDINGWINDOW:4:20). After reviewing the read length histograms generated with bbmap (v38.82) [30], only reads with a minimum length of ≥ 50 bp were kept [4a-3A: 49,480,787 paired end reads kept (66.87%); 4a-3B: reads kept 48,149,543 (67.01%)]. Trimmed reads were mapped to the *An. gambiae* PEST genome version AgamP4 (Vectorbase; https://vectorbase.org) using BWA mem (v0.7.17) [31]. Samtools (v1.11) [32, 33] was used to fix mate pair issues, remove reads that mapped singly or not with their mate (4a-3A: 95.61% kept; 4a-3B: 94.29% kept), remove duplicate reads [4a-3A: 4,379,538 reads removed (4.63%); 4a-3B: 6,682,812 reads removed (7.36%)] and filter by mapping quality score filtering (> 10) (4a-3A: 39,595,140 paired end reads kept; 4a-3B: 37,148,035 reads kept). Mapping coverage was determined using samtools coverage with default parameters [32, 33].

#### SNP and INDEL variant analysis

Prior to GATK variant analysis [34], the following read groups were added using PICARD AddOrReplaceReadGroups [35]: 4a-3A: RGID = 4a-3A, RGLB = TruSeq_Nano, RGPL = ILLUMINA, RGPU = S1, RGSM = 4a-3A_DNA; 4a-3B: RGID = 4a-3B, RGLB = TruSeq_Nano, RGPL = ILLUMINA, RGPU = S2, RGSM = 4a-3B_DNA. Both the 4a-3A and 4a-3B bam files were variant called using the GATK HaplotypeCaller program with heterozygosity, heterozygosity-stdev and indel-heterozygosity values set based on the GATK protocol used by the Ag1000G project [36]. The resulting GVCF files were combined into a database with GenomicsDBimport and then joint genotyped with GenotypeGVCFs using the same parameters specified for HaplotypeCaller above. In total 2,344,505 SNPs and 516,815 INDELs were identified using the GATK variant calling protocol. These totals include SNPs and INDELs where both 4a-3A and 4a-3B are different from the AgamP4 genome assembly and SNPs and INDELs where one cell line matches the AgamP4 genome assembly, and the other cell line has a distinct genotype.

SNP and INDEL variants were hard filtered separately using VariantFiltration, SNPs by QUAL, SOR, FS, MQ, MQRankSum & ReadPosRankSum and INDELs by QUAL, FS & ReadPosRankSum using the recommended cut-off values present in the GATK documentation. Further filtering was performed using vcftools (v0.1.16) to remove all variant sites containing any missing data [37]. Minimum allele balance (MIN_AB 0.2), minimum depth (MIN_DP 10) and minimum genotype quality (MIN_GQ 20) were filtered using PICARD FilterVcf [35]. Only biallelic indels and SNPs were considered for further analysis (multiallelic SNPs: *n *= 8780; multiallelic INDELs: *n* = 8993 were removed). Mode mean depth for both SNPs and INDELs was calculated using vcftools and the Tidyverse R package, and a maximum mean depth per variant site was set at 2 × mode mean depth for both variant types [37, 38]. After all filtering steps, 1,808,124 SNPs (77.12%) and 410,644 INDELs (79.46%) remained. SNPs and indels fixed relative to the AgamP4 genome assembly were identified using GATK SelectVariants [34]. Venn diagrams of fixed INDELs and SNPs were made using the Vennerable R package [39].

Analysis of the distribution of SNPs and indels across the AgamP4 genome assembly was performed using χ^2^ statistical tests in Graphpad Prism version 9.3.1. A sliding window analysis (window size 10,000 bp, step 2500 bp) was performed for each chromosome arm using the windowscanr R package [40]. Further analysis of the 2R chromosome arm was also performed, examining the distribution of SNPs and INDELs across the known inversion regions using Graphpad Prism.

Genetic variation of the six prophenoloxidase (PPO) genes, previously identified as constitutively expressed in 4a-3B but not in 4a-3A [5], was examined using the Integrative Genomics Viewer (IGV)(v2.12.3) [5, 41–43].

#### Inversion typing assays

Molecular assays for the 2Rb, 2Rj and 2La inversions were performed on genomic DNA extracted from 4a-3A and 4a-3B cells following published methods [44–47]. In silico inversion typing was performed using Compkaryo for the 2Rb, 2Rc, 2Rd, 2Rj and 2Ru inversions [48].

#### Species typing assay

Genomic DNA was extracted from 4a-3A, 4a-3B, Ag55 and Sua4.0 cells as described above. Molecular species typing was performed on the genomic DNA isolated from 4a-3A, 4a-3B, Ag55 and Sua4.0 cell lines using the SINE200 assay [49]. In silico examination of the S200 X6.1 INDEL site was performed using Integrative Genomics Viewer (IGV) [41–43]. In silico species typing using 20 previously identified variable SNPs present across the 2L, 3L and X speciation islands was also performed [50–55]. In addition to in silico typing of 4a-3A and 4a-3B cell lines, the ancestry of the PEST AgamP4 genome assembly was examined. The proportion of *An. gambiae* ancestry was calculated by assigning a value of 1 to SNPs of *An. gambiae* origin, a value of 0 to SNPs of *An. coluzzii* origin and a value of 0.5 for heterozygotes and then averaging the scores across the 20 SNPs [51]. However, because the PEST reference genome is not uniformly heterozygous, estimates of *An. gambiae* ancestry may be inaccurate.

#### Molecular assay to distinguish *Anopheles* cultured cell lines

The differentially fixed indels between 4a-3A and 4a-3B cells were examined to identify candidates for use in a molecular assay capable of differentiating the cell lines. The following criteria were used to prioritize indels for testing: (1) distinct predicted genotype between 4a-3A and 4a-3B cells (82,225 indels); (2) indel has a predicted size allowing for ease of PCR and agarose gel detection (131 indels are > 79 bp); (3) a BLAST search against the AgamP4 genome assembly returns a single location for a deletion or zero matches for an insertion relative to the PEST AgamP4 genome assembly; (4) indel sequence is not low complexity (i.e. not comprised of microsatellites or other repeats); (5) target indels are not flanked by other nearby predicted indels; (6) target indels are spatially distributed throughout the genome. Primers for the first five candidate indel regions that met the above criteria were designed with Primer3 (https://primer3.ut.ee/) (Additional file 1: Table S1) [56–58]. Indels were named by chromosome arm and the left-most position in the AgamP4 genome assembly (indel located on 2R at 25,670,547 bp was named 2R.25670547). Indel regions were amplified in individual PCR reactions containing 1× PCRBIO Ultra mix (Genesee Scientific), 20 ng cell line genomic DNA and 500 nM primers with cycling conditions of an initial denaturation step at 98.0 °C for 30 s, followed by 35 cycles of 98.0 °C for 10 s, 65 °C for indel 3L.40012707 or 60 °C for the other four indels tested (2R.25670547, 3R.11632056, 3R.11788474, 3R.11809836) for 30 s and 72.0 °C for 45 s with a final extension at 72.0 °C for 10 min. All products were run on a 2% agarose gel and visualized. To size bands observed on a gel 100-bp ladder (New England Biolabs) and GeneRuler 1-kb Plus DNA Ladder (Thermofisher) were used. In addition to assaying indel size in genomic DNA isolated from 4a-3A and 4a-3B cells, assays were also performed on genomic DNA isolated from Ag55 and Sua4.0 cell lines.

Following an initial screen, three of the tested indel regions, 2R.25670547, 3R.11788474 and 3R.11809836, were selected because of the distinct molecular fingerprint observed for each of the four cell lines tested (the molecular fingerprint of indels 3L.40012707 and 3R.11632056 were redundant with that of indel 2R.25670547). PCR products were Sanger sequenced to verify the indels detected from whole genome sequence analysis (Molecular Cloning Laboratories, MCLAB, https://www.mclab.com/). Due to the presence of heterozygous indels, PCR amplicons of the 3R.11788474 and 3R.11809836 indel regions amplified from Ag55 genomic DNA were cloned prior to sequencing. Sequences of the three indel regions in each of the four cell lines are in Additional file 1.

In addition to serving as authentication tools for cells lines, the same indels were assayed for their ability to detect culture contamination of 4a-3A and 4a-3B cells. Genomic DNA isolated from 4a-3A and 4a-3B cell lines was mixed at ratios of 1:9, 1:4, 1:2, 1:1, 2:1, 4:1 and 9:1, and 20 ng of these DNA mixtures was used as template to amplify the 3R.11788474 indel. PCR products were run on a 2% agarose gel and visualized.

### Cytogenetics methods

#### Colcemid treatment of cells

Cell lines 4a-3A and Sua4.0 were cultured as described above in 250-ml flasks and were passaged at a 1:5 dilution to reach monolayer formation with a cell density of 50%–60%. Cells were treated for 1.5 h at 27 °C with 0.1 µg/ml colcemid (MilliporeSigma, St. Louis, MO, USA; catalog no. 10295892001) by adding 100 µl of 10 ug/ml colcemid to 10 ml of cell culture medium.

#### Cell fixation for cytogenetics

Cells were suspended in a culture flask with a spatula, transferred to a 10-ml centrifuge tube and centrifuged at 1300 rpm for 7 min. The supernatant was carefully removed, and the pellet was resuspended and treated with 2 ml of hypotonic solution (0.075 M KCl) for 25 min at 37 °C. After incubation, the cells were prefixed by adding 100 µl of fresh Carnoy’s fixative solution (methanol: acetic acid, 3:1) for 5 min at RT, the suspension was centrifuged at 13,000 rpm for 7 min, and the pellet was disaggregated and fixed in 1 ml of fresh Carnoy’s fixative solution without pipetting. The cell suspension was kept on ice for 20 min before making chromosomal preparations.

#### Chromosome preparation

Preparations were made by the dropping technique. The cell pellet was gently and thoroughly pipetted, centrifuged and fixed in fresh Carnoy’s fixative solution twice. Each time cells were kept on ice for 10 min for fixation. After the last centrifugation step, the pellet was dissolved in 200 µl of Carnoy’s fixative solution to make the suspension slightly cloudy but transparent. The glass slide was rinsed with sterile water before adding cells. Three to four drops of fixed cells were “dripped” on the clean wet slide from a 5 cm distance between slide and pipette tip. The slides were kept on ice for 2–3 min in upside-down position and then air-dried completely.

#### Chromosome staining and imaging

Chromosome preparations were counterstained with a ProLong Gold antifade reagent with DAPI (Life Technologies, Carlsbad, CA, USA) and kept in the dark for at least 2 h before visualization. Chromosome images were obtained using a Zeiss AXIO fluorescent microscope with an Axiocam 506 mono digital camera (Carl Zeiss AG, Oberkochen, Germany) at 1000 × magnification.

## Results

### Genomic distribution of variants

Analysis of whole genome sequencing on *Anopheles* 4a-3A and 4a-3B cell lines was used to detect SNPs and indels variable between these two cell lines. The 4a-3A paired-end reads mapped to the *An. gambiae* reference genome assembly, AgamP4, with chromosome arm percent coverage of 91.1 at a mean depth of 35.5× (arm 2R), 94.8 at 40.0× (arm 2L), 94.0 at 41.9× (arm 3R), 91.8 at 36.3× (arm 3L) and 94.2 at 39.9× (arm X). The 4a-3B paired-end reads mapped to the AgamP4 reference with percent coverage of 90.3 at a mean depth of 35.6× (2R), 94.7 at 36.7× (2L), 94.1 at 38.0× (3R), 89.2 at 33.7× (3L) and 94.2 at 39.8× (X). Genome-wide coverage for 4a-3A and 4a-3B was 93.2% and 92.5% with an average mean read depth of 38.7× and 36.8×, respectively.

**Fig. 1:**
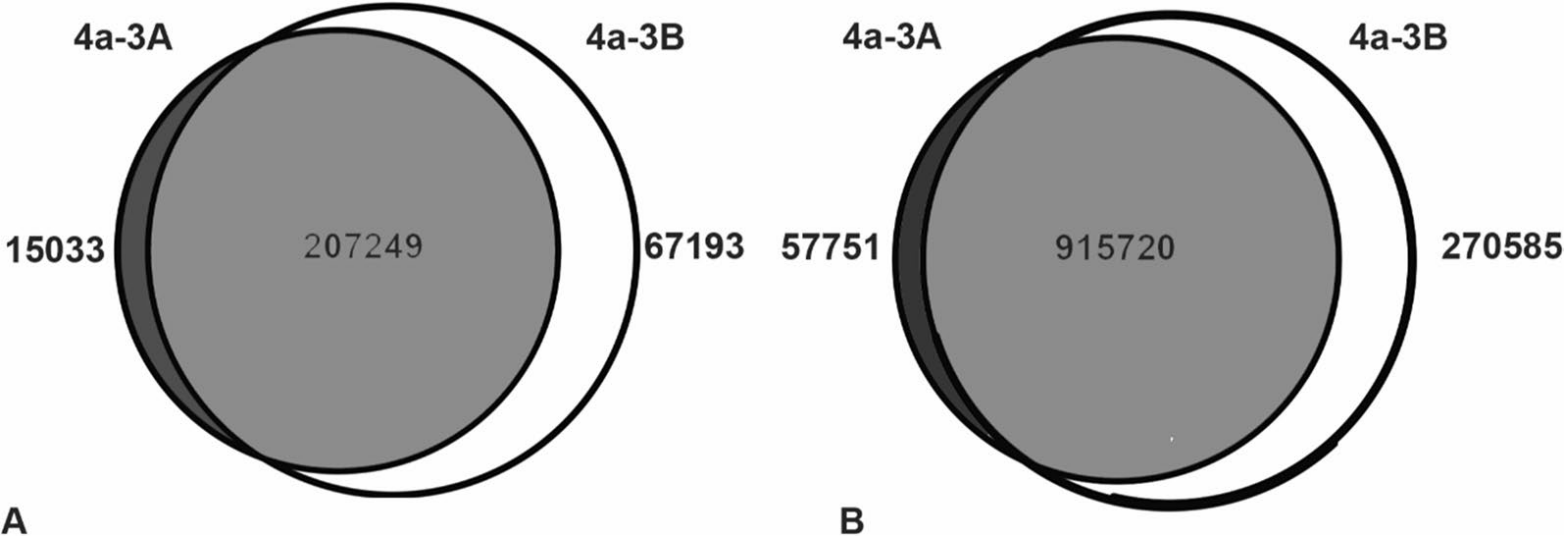
Fixed variants present in 4a-3A and 4a-3B cells. **A** Of the 289,485 fixed indels identified in 4a-3A and 4a-3B cells, 72% of variants are present in both cell lines, 23% are unique to 4a-3B, and the remaining 5% are unique to 4a-3A. **B** Of the 1,244,056 fixed SNPs identified in 4a-3A and 4a-3B, 74% of variants are present in both cell lines, 21% are unique to 4a-3B, and the remaining 5% are unique to 4a-3A

Following variant calling and filtering, a total of 1,808,124 SNPs and 410,644 indels were identified with an average mean read depth per variant site of 45.7× and 39.8×, respectively. Fixed variants relative to the AgamP4 reference were identified as shown in Fig. 1. A total of 289,475 fixed indels were identified with the majority of these, 207,249 (72%), having the same genotype in both 4a-3A and 4a-3B cells, but with each cell line distinct from the AgamP4 genome assembly, and the minority of indels, 15,033 (5%) and 67,193 (23%), were unique to 4a-3A and 4a-3B cells, respectively (Fig. 1A). A similar pattern was observed for the 1,244,056 fixed SNPs; again the majority of SNPs, 915,720 (74%), were present in both cell lines but distinct from the AgamP4 genome assembly and the minority of SNPs, 57,751 (5%) and 270,585 (21%) were unique to 4a-3A and 4a-3B cells, respectively (Fig. 1B). At both the SNP and indel level, the 4a-3B cell line was about 4 × more divergent from the *An. gambiae* PEST genome compared to the 4a-3A cell line.

**Fig. 2:**
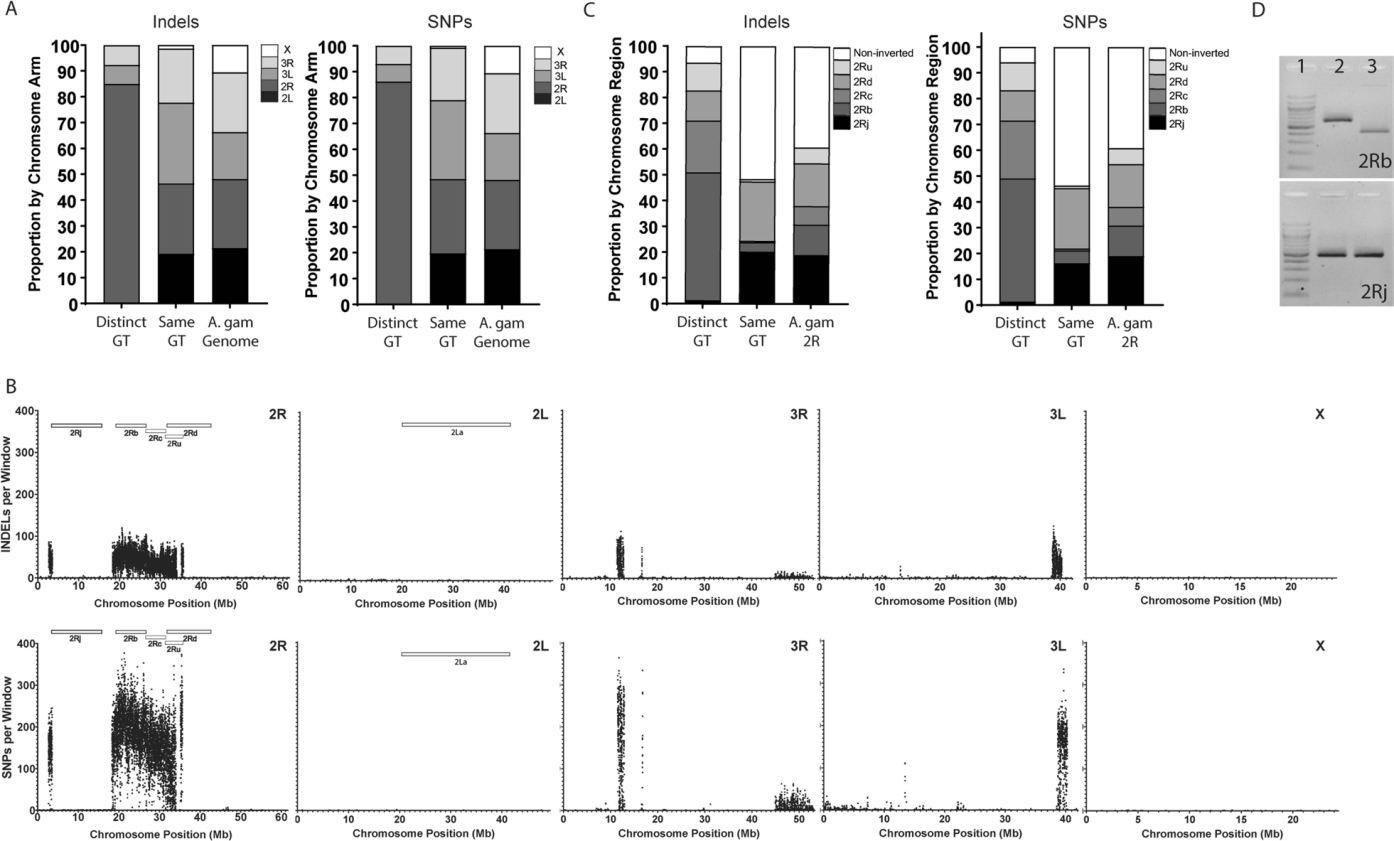
Genetic variants differentiating 4a-3A and 4a-3B cells are clustered on the 2R chromosome. **A** The distribution of fixed indels (left) and SNPs (right) is distinct from the total genome composition of the chromosome arms, all pairwise χ^2^ *P*-values < 0.0001. Distinct GT: variants have a distinction between the 4a-3A and 4a-3B cell lines. Same GT: variants have the same genotype in the 4a-3A and 4a-3B cell lines but each are distinct from the *An. gambiae* AgamP4 reference genome assembly. **B** Distribution of cell line Distinct indels (top) and SNPs (bottom) across the *An. gambiae* AgamP4 reference genome assembly. Window size of 10,000 bp with a step size of 2500 bp. *Position of the three indels used in the diagnostic assay described in Fig. 4. **C** The distribution of fixed indels (left) and SNPs (right) is distinct from the overall composition across the 2R chromosome arm, all pairwise χ^2^ *P*-values < 0.0001. **D** Molecular diagnostic for the 2Rb (top) and 2Rj (bottom) chromosome inversions using published methods. Lane 1: 100-bp ladder, lane 2: 4a-3A, lane 3: 4a-3B. Expected size for 2R + ^j^ is 494 bp and for 2Rj is 253 bp (47) and for 2R + ^b^ is 630 bp and for 2Rb is 429 bp

The distributions of SNP and indel variants fixed alternately in the two cell lines [Distinct Genotype (GT)] and the variants fixed similarly in both cell lines, but having a genotype distinct relative to the AgamP4 genome assembly (Same GT), were significantly different across the genome from the expectation if SNPs and indels occurred randomly across the chromosome arms (Fig. 2A; Distinct GT vs. the AgamP4 genome assembly and Same GT vs. the AgamP4 genome assembly χ^2^ *P*-values < 0.0001). Same GT variants are present across all chromosome arms with less apparent clustering than the Distinct GT variants with the exception of an underrepresentation of variants on X (0.72% of SNPs and 1.33% of indels) and an overrepresentation on 3L (30.6% of SNPs and 31.3% of indels). Distinct GT variants clearly clustered on 2R with 86.2% of Distinct GT SNPs and 84.8% of Distinct GT indels identified on 2R (Fig. 2A). Many Distinct GT variants occurred in the regions of the 2Rb, 2Rc and 2Ru inversions. Sliding window analysis of the distribution of the Distinct GT variants also revealed clustering of variants in additional short chromosomal regions of 2R, 3R and 3L (Fig. 2B). In contrast, Distinct GT variants were underrepresented on 2L and X with only 0.003% distinct SNPs and 0.12% distinct indels identified on 2L and 0.01% distinct SNPs and 0.038% distinct indels identified on X. While most of the 2R variants were present in clusters overlapping the known 2Rb, 2Rc and 2Ru inversion regions, the 2R 2.66–3.56 Mb variant cluster and the clusters on 3R and 3L could represent novel micro-inversion regions distinct between 4a-3A and 4a-3B cells (Fig. 2B). Further examination of the 2R 2.66–3.56 Mb cluster revealed three distinct regions, 833 variants in 4a-3B cells distinct from the AgamP4 genome assembly followed by 5767 distinct variants in 4a-3A cells and then another 9116 distinct variants in 4a-3B cells. Of the remaining clusters identified on 2R, 3R and 3L, three contained a majority of variants distinct in 4a-3B cells (2R 18.28-34.042 Mb, 3L 38.709-40.29 Mb, 3R 16.76-16.795 Mb) and two contained the majority of variants distinct in 4a-3A cells (2R 35.1525-35.645 Mb, 3R 11.515-12.915 Mb).

### Genetic variation observed surrounding prophenoloxidase genes

Distinct from 4a-3A cells, 4a-3B cells were shown to constitutively express six prophenoloxidase genes, PPO1-6 [5]. The PPO1 gene is located within the variant cluster 2R 18.28-34.042 Mb within the known 2Rc inversion and carries variants distinguishing 4a-3B and 4a-3A cells. Relative to 4a-3A cells, 4a-3B cells contain 21 noncoding indels (20 intronic, 1 in the 3’–UTR), 134 noncoding SNPs (2 in the 5’-UTR, 122 intronic and 10 in the 3’-UTR) and 13 synonymous coding SNPs (4 in Exon 2, 4 in Exon 3, 2 in Exon 4, 3 in Exon 5) within the PPO1 gene region. Using a genome-wide catalog of transcriptional enhances [9], variation was also examined in two predicted enhancer regions, 2R:27996659-27997160 and 2R:28016526-28017040, close to PPO1. Examining sequence difference across these two candidate enhancers, 4a-3B cells had 0 indels and 2 SNPs relative to 4a-3A cells for the enhancer at 2R:27996659-27997160 and 3 indels and 13 SNPs in the enhancer located at 2R:28016526-28017040. The remaining five prophenoloxidase genes, PPO2-6, contained no variants distinguishing 4a-3A cells from 4a-3B cells. Three additional predicted enhancer regions were identified near PPO genes 2–6, one near PPO2 [1] and two near the PPO3/PPO4/PPO6 cluster on 2L. None of these three enhancer regions contained variants differentiating the cells lines from one another. Thus, the two cell lines display distinct genetic variants in and near the PPO1 gene, including in enhancers. Further work will be required to determine the significance of these polymorphisms for control of PPO1 expression.

### 2R inversion typing of 4a-3A and 4a-3B cells

In addition to using whole genome sequences to detect chromosomal inversions, molecular and in silico karyotyping assays were used to confirm inversion status. Molecular diagnostics indicated that both 4a-3A and 4a-3B cells (Fig. 2D) were homozygous for the 2R + ^j^ form of the 2Rj inversion. While 4a-3A cells were homozygous for the 2R + ^b^ form of the 2Rb inversion, 4a-3B cells were homozygous for the 2Rb form [45, 47]. Both cell lines were homozygous for the 2L + ^a^ form of the 2La inversion (Additional file 1: Fig. S1) [46]. In silico karyotyping yields prediction for 2Rb and 2Rc inversion alleles in both cell types [48]. Compkaryo identified 290 diagnostic SNPs (83.1% of total diagnostic 2Rb SNPs utilized by Compkaryo) in our dataset for 2Rb with 4a-3A identified as 2R + ^b^ (b + : 283; b: 7) and 4a-3B as 2Rb (b + : 1; b: 289), consistent with both the molecular karyotyping results and the whole genome sequence analysis (Fig. 2B). Fifty-nine 2Rc diagnostic SNPs (49 *An. coluzzii* and 10 *An. gambiae*; 55.1% of diagnostic 2Rc SNPs) predicted 4a-3A as 2R + ^c^ (c + : 58; c: 1) and 4a-3B as 2Rc (c + : 1; c: 58), again consistent with the whole genome analysis (Fig. 2B). The 2Rj and 2La inversions contained one and zero Compkaryo diagnostic SNPs, respectively, and as a result were not in silico karyotyped in 4a-3A and 4a-3B cells, though both were molecularly typed (Fig. 2D and Additional file 1: Fig. S1). This lack of variant sites for the 2La and 2Rj inversions likely stemmed from the fact that their inversion status did not differ across cell lines (Fig. 2D and Additional file 1: Fig. S1). The 4a-3A and 4a-3B sequence data identified only six Compkaryo diagnostic SNPs (4.1% of total) in the 2Rd inversion and only nine Compkaryo diagnostic SNPs in the 2Ru inversion (5.1% of the total). Given the low number of variant sites, accurate prediction of karyotype for these two inversions was also not reliable. Taken together, the in silico karyotyping yielded results consistent with whole genome sequence analysis and molecular karyotyping and yielded the following karyotypes across six known inversions: 4a-3A carries the following karyotype inversions 2R + ^j^, 2R + ^b^, 2R + ^c^ and 2L + ^a^ while 4a-3B cells were 2Rj, 2Rb, 2Rc and 2L + ^a^ with 2Rd and 2Ru inversion status remaining undefined.

### Species typing of 4a-3A and 4a-3B cells

**Fig. 3:**
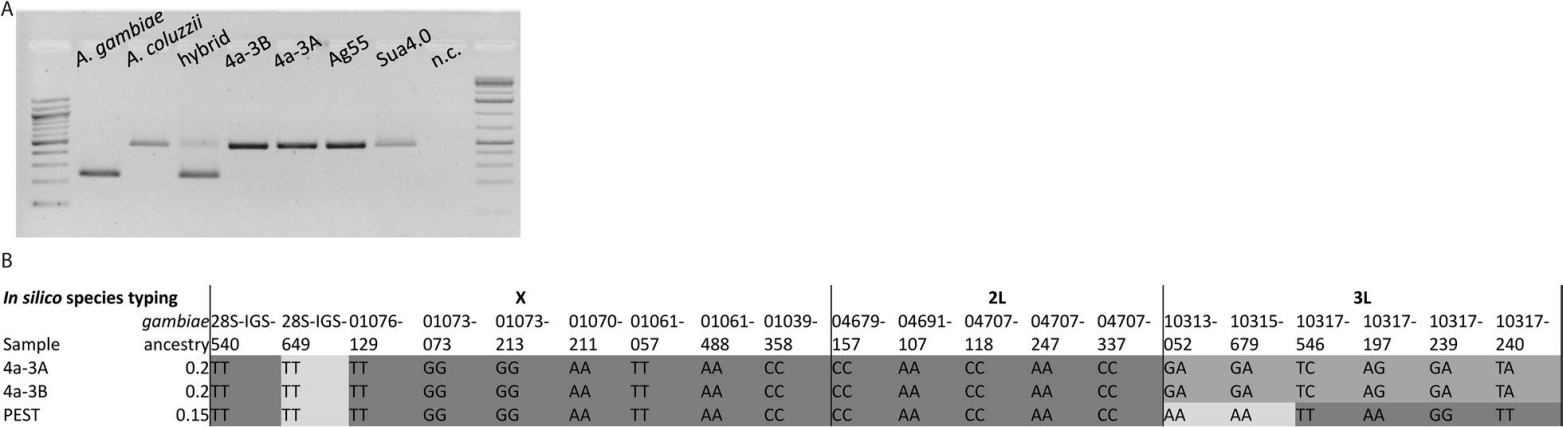
Molecular and in silico species typing of *Anopheles* cell lines indicates 4a-3A and 4a-3B are primarily *An. coluzzii* in origin. **A** The S200 X6.1 assay [49] molecular species diagnostic was run on genomic DNA isolated from cell lines. Lane 1 contains a 100-bp ladder; lanes 2 and 3 show amplified products from known *An. gambiae* and *An. coluzzii* samples, respectively. Lane 4 is amplified product from an equal volume mixture of the PCR template used in lanes 2 and 3 and thus represents a species hybrid. Lanes 5–8 show amplified product from 4a-3B, 4a-3A, Ag55 and Sua4.0 genomic DNA, respectively. Expected band sizes are 479 bp for *An. coluzzii* and 249 bp for *An. gambiae*. Lane 9 is a no template control, and lane 10 contains GeneRuler 1-kb Plus DNA Ladder. (**B**) In silico species typing performed following a previously published method [51]. SNP names follow the naming scheme provided by Lee et al. 2014 [51] with the last five digits of the AGAP gene name followed by the position of the SNP in the coding sequence of the gene (e.g. 01706-129 is a SNP at position 129 in AGAP001706). The second column of the table gives the proportion of *An. gambiae* ancestry ranging from 0 to 1 with 0 indicating complete *An. coluzzii* ancestry and 1 indicating complete *An. gambiae* ancestry. Gray shading indicates the degree of *An. gambiae* ancestry; dark gray 100% *An. coluzzii*, light gray 100% *An. gambiae* and medium gray species hybrids. In silico species typing was also done for the AgamP4 genome assembly; however, this may be an over- or underestimate of *An. gambiae* ancestry because genome assembly lacks heterozygosity

Previous literature typically described 4a-3A, 4a-3B, Ag55 and Sua4.0 cell lines as *An. gambiae* in species origin. Molecular typing using the SINE200 diagnostic assay [49] indicated that all four lines were derived from *An. coluzzii* (Fig. 3A). In silico species typing using 20 previously identified SNPs on the X, 2L and 3L speciation islands indicated that both 4a-3A and 4a-3B cell lines had an *An. gambiae* ancestry of only 20% with an 80% *An. coluzzii* ancestry(Fig. 3B) [50–55]. The genotypes of the 20 SNPs were identical between the two cell lines with 1 *An. gambiae* SNP, 6 heterozygous SNPs and 13 *An. coluzzii* SNPs identified. This indicates that not only was each cell line 20% *An. gambiae*, but, at least at the level of resolution afforded by this assay, the same portions of the 4a-3A and 4a-3B genomes were derived from *An. gambiae* in each case. In silico species typing was also performed on the PEST reference strain as represented by the AgamP4 reference assembly, which indicated PEST ancestry of 15% *An. gambiae* (85% *An. coluzzii*), with 3 *An. gambiae* SNPs and 17 *An. coluzzii* SNPs (Fig. 3B). As the *An. gambiae* genome assembly does not contain heterozygous sequence, this estimate for PEST ancestry should be regarded as an approximation.

### Authentication assay detected distinct molecular fingerprints of four anopheline lines

**Fig. 4:**
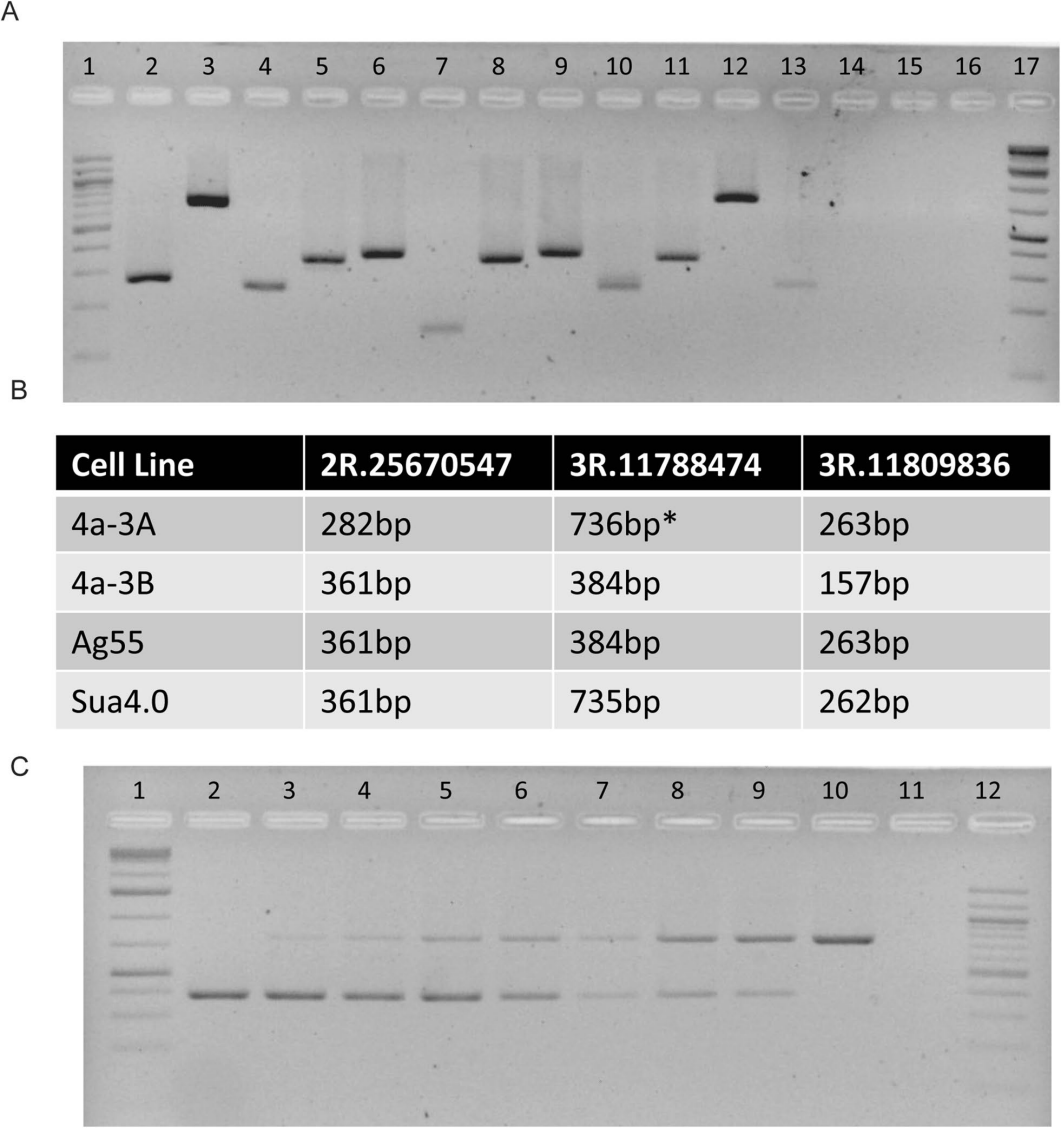
Molecular assay to differentiate 4a-3A, 4a-3B, Ag55 and Sua4.0 cell lines. **A** Molecular fingerprints of three PCRs amplifying indel regions in 4a-3A (lanes 2–4), 4a-3B (lanes 5–7), Ag55 (lanes 8–10), Sua4.0 cells (lanes 11–13) and no PCR template controls (lanes 14–16); 100-bp ladder (lane 1), indel 2R.25670547 (lanes 2, 5, 8, 11, 14), indel 3R.11788474 (lanes 3, 6, 9, 12, 15), indel 3R.11809836 (lanes 4, 7, 10, 13, 16) and 1-kb Plus DNA Ladder (lane 17). **B** Table of expected PCR product sizes for 2R.25670547, 3R.11788474 and 3R.11809836 for each of the cell lines based on Sanger sequencing results. *Predicted size based on whole genome sequencing was 532 bp for 3R.11788474 in 4a-3A genomic DNA; however, upon Sanger sequencing an additional indel of 204-bp size was discovered following the predicted 148-bp indel resulting in an amplicon size of 736 bp. All sizes were verified by Sanger sequencing. **C** Detection of contamination by indel assay. The 3R.11788474 indel assay is able to detect contaminating genomic DNA present at 10% of the total PCR template input. Lane 1 GeneRuler 1-kb Plus DNA Ladder; lane 2: 20 ng 4a-3B gDNA; lane 3: 18 ng 4a-3B gDNA and 2 ng 4a-3A gDNA; lane 4: 16 ng 4a-3B gDNA and 4 ng 4a-3A gDNA; lane 5: 13.3 ng 4a-3B gDNA and 6.6 ng 4a-3A gDNA; lane 6: 10 ng 4a-3B gDNA and 10 ng 4a-3A gDNA; lane 7: 6.6 ng 4a-3B gDNA and 13.3 ng 4a-3A gDNA; lane 8: 4 ng 4a-3B gDNA and 16 ng 4a-3A gDNA; lane 9: 2 ng 4a-3B gDNA and 18 ng 4a-3A; lane 10: 20 ng 4a-3A gDNA, lane 11: control, no PCR template, lane 12: 100-bp ladder

The anopheline lines 4a-3A, 4a-3B, Ag55 and Sua4.0 are morphologically indistinguishable. While the molecular diagnostic for the 2La inversion [46] allows differentiation of Sua4.0 from the other three lines, because Sua4.0 is fixed for 2La and the other lines are fixed for 2L + ^a^ (Additional file 1: Fig. S1), there is no current assay that allows 4a-3A, 4a-3B and Ag55 cell lines to be distinguished from each another. Therefore, five indel regions with a distinct genotype between 4a-3A and 4a-3B cells were selected (2R.25670547, 3L.40012707, 3R.11632056, 3R.11788474 and 3R.11809836) and tested for the presence of diagnostic genotypes across all four cell lines (Fig. 4A). The molecular fingerprints of indels 3L.40012707 and 3R.11632056 were redundant with that of indel 2R.25670547; thus, only 2R.25670547, 3R.11788474 and 3R.11809836 were used in the authentication assay.

The 2R.25670547 indel is a 79-bp deletion relative to the AgamP4 genome assembly in 4a-3A cells alone while the 3R.11788474 indel is a 148-bp insertion relative to the AgamP4 genome assembly in both 4a-3A and Sua4.0 cells (Fig. 4B). The 3R.11809836 indel is a 106-bp insertion relative to the AgamP4 genome assembly in 4a-3A, Ag55 and Sua4.0 cells (Fig. 4B). When the PCR products were sequenced for these three indel regions, all three displayed a small number of SNPs that were also distinct between the four cell lines (Additional file 1). In addition, sequencing of the 3R.11788474 indel resulted in the identification of an additional indel relative to the AgamP4 genome assembly in 4a-3A and Sua4.0 cells which was 204 bp in length and 91 bp downstream of the 148-bp insertion originally detected in the variant analysis. Identification of these additional indels relative to the AgamP4 genome assembly is not surprising as finer level discrimination of indel events is expected from Sanger sequencing data as the short-read Illumina sequence has limited power for indel resolution. The Ag55 cell line was found to contain two distinct alleles in the 3R.11788474 and 3R.11809836 indel PCR products; in both cases, these alleles differentiated at multiple SNP and small indel locations, but these small indels were not differentiated using the PCR and gel conditions described in the methods and depicted in Fig. 4A. When tested on early and late cell passages of 4a-3A and Sua4.0 cells, the authentication assays gave identification results and therefore appear robust to passage number (Additional file 1: Fig. S2). Therefore, the 2R.25670547, 3R.11788474 and 3R.11809836 indel regions produced a viable option for a cell line authentication assay as together they allow the 4a-3A, 4a-3B, Ag55 and Sua4.0 lines to be distinguished based on unique molecular fingerprints. In addition to the misidentification of cell lines, cross contamination of one cell line with another is also an important problem that can skew experimental results. Therefore, the indels selected for the authentication assay were examined to determine whether one of them could be used as a marker for cross contamination between the 4a-3A and 4a-3B cell lines. The 3R.11788474 indel was able to detect contaminating genomic DNA at ≥ 10% of the total PCR template input for the case of contaminated 4a-3A and 4a-3B cells (Fig. 4C).

### Cytology and determination of cell ploidy

**Fig. 5:**
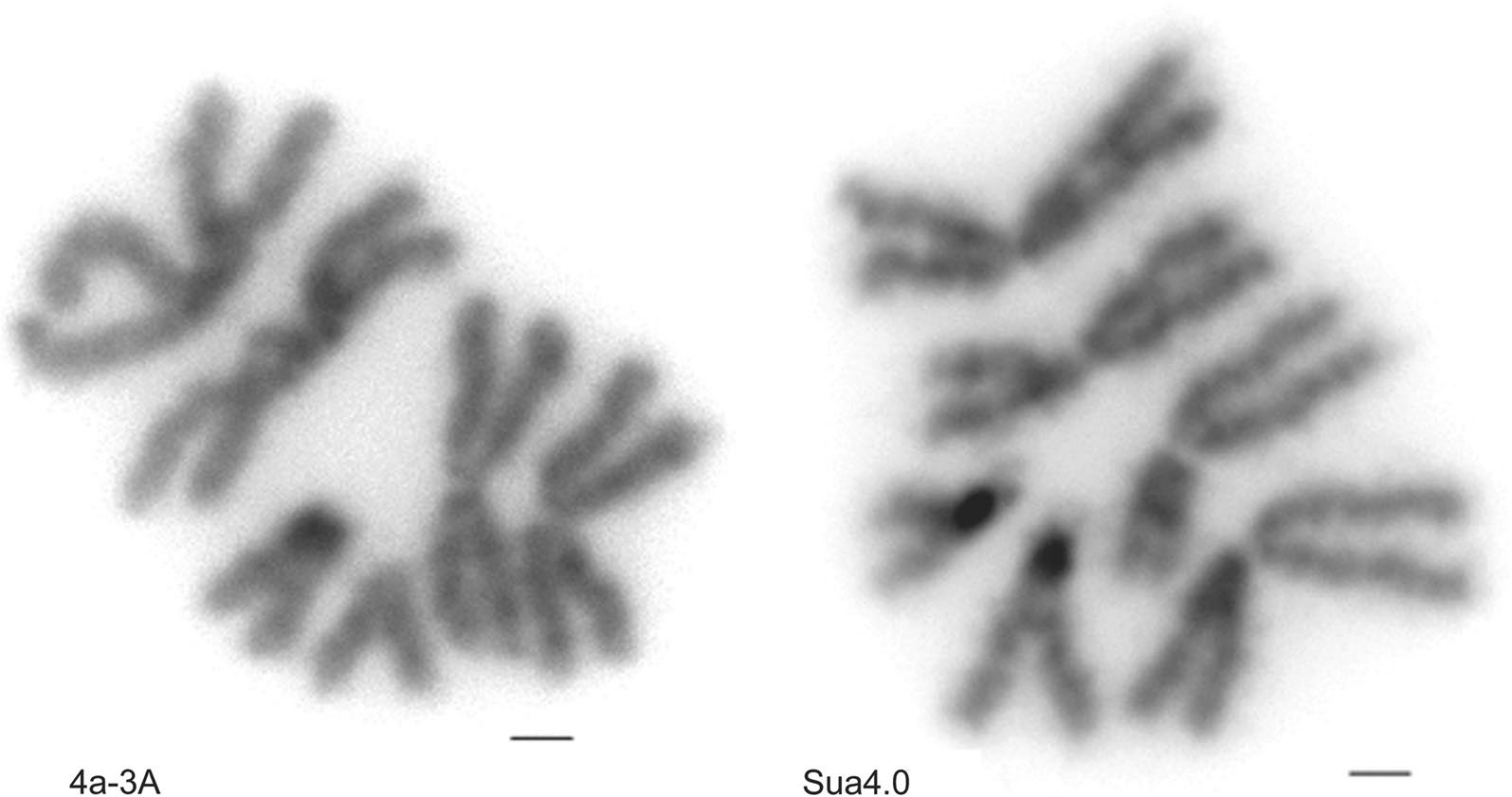
4a-3A and Sua4.0 cells are diploid. Representative images of chromosome preparations depicting the 2 autosomes (2 and 3) and the X chromosome. Both cell lines examined are female in origin and > 95% diploid. Chromosomes are shown in gray color. Scale bar: 1 µm

Cells from the 4a-3A and Sua4.0 cell lines were cytologically examined for ploidy levels. 4a-3A is a female cell line, as shown by the presence of a pair of X chromosomes, and of 331 4a-3A cells examined, 95% (*n* = 314) were diploid. Visual examination of the cytological preparations indicated that the homologous chromosomes in the majority of 4a-3A cells have similar lengths, suggesting that there is little autosomal rearrangement (Fig. 5). Sua4.0 is also a female cell line. Of 124 Sua4.0 cells examined, again 95% (*n* = 118) were diploid. In Sua4.0 cells, all homologous chromosomes appear to have different lengths consistent with the presence of some rearrangements. For both cell lines, 5% of observed cells were tetraploid, which can be an artifactual result of cell treatment with colcemid [59, 60].

## Discussion

Whole genome sequencing of two *Anopheles* cell lines highlighted SNP and indel variation unique to 4a-3A and 4a-3B cell lines. Based on this variation, an identification and authentication assay was developed that can distinguish these two cell lines and also two other commonly used mosquito cell lines, Ag-55 (Mos55) and Sua4.0. Thus, with three PCR reactions using identical cycling conditions, an investigator can distinguish among four *Anopheles* cell lines. This indel-based PCR assay allows for clear differentiation on an agarose gel, is easy to execute and requires no specialty laboratory equipment or reagents. This assay serves not only to identify cell lines but also as a tool to detect cell line contamination.

The initial report of the 4a-3A and 4a-3B cell lines (5), as well as many subsequent publications utilizing them, refers to them as originating from *An. gambiae*. The current genomic assessment clearly demonstrates an origin more closely rooted in *An. coluzzii*, with fewer than 20% of species diagnostic SNPs consistent with *An. gambiae* origin.

Similar identification and authentication tools should be developed and utilized for commonly used anopheline mosquito colonies, as contamination and mislabeling of colonies can lead to spurious results. The Malaria Research and Reference Reagent Resource Center used an authentication matrix to distinguish among genetically similar *Anopheles* colonies [61]. Frequently, cell lines and mosquito colonies are exchanged between researchers without validation, verification or colony standardization. As gene drives and CRISPR-modified cell lines and mosquito colonies become more common, the need to determine colony purity and detect contamination will only increase [62]. Experimental rigor and reproducibility will benefit from better community standards for mosquito cell line and colony authentication, especially as results from insect infection studies are compared across research facilities [63].

One notable difference between the 4a-3A and 4a-3B cell lines is their expression of six prophenoloxidase genes, PPO1-6, which 4a-3A cells do not express, while 4a-3B cells express all six PPO genes constitutively [5]. Current work indicates that only one of these genes, PPO1, is genetically variable between 4a-3A cells and 4a-3B cells, and the coding variation segregating between the cell lines is all synonymous. Using our recently reported genome-wide catalog of transcriptional enhancers [9], genetic variations in candidate enhancers near all six PPO genes were examined. Most non-coding variation lies in enhancers near the PPO1 gene, with most occurring in a predicted enhancer located at 2R:28016526-28017040, just 1665 bp upstream of the PP01 gene. The current work provides the resources to allow more confident exploitation by the community of these cell lines to research biological mechanisms, such as prophenoloxidase expression and others.

Cytogenetic analysis demonstrates that both the 4a-3A and Sua4.0 cell lines are diploid. Knowledge of ploidy is a relevant and important consideration for experiments involving genetic modification via CRISPR or similar technologies. While greater ploidy does not preclude genetic modification, using diploid cell lines simplifies the experimental problem.

## Conclusion

Genome characterization of *Anopheles* cell lines 4a-3A and 4a-3B reveals that both are diploid cell lines of female origin with the bulk of the genetic variation between them resulting from a fixation of the 2Rb, c and u inversions within the 4a-3B cell line. Contrary to previous reports of an *An. gambiae* species origin, these cell lines are primarily of *An. coluzzii* ancestry. An authentication assay based on PCR amplification of three variable indels is capable of distinguishing not only 4a-3A and 4a-3B, but also Ag55 and Sua4.0 cell lines.

## Supplementary Information

**Additional file 1: Table S1.** Primer sequences for indels selected for cell line diagnostic. Fasta file of indel region sequences. Sequence of the three diagnostic indel regions, 2R_25670547, 3R_11788474 and 3R_11809836, in each of the four tested cell lines. 2La inversion. **Figure S1.** Molecular karyotyping of the 2La inversion in four cell lines. Sua4.0 cells are fixed for the 2La inversion, and 4a-3A, 4a-3B and Ag55 cells are fixed for 2L + a. Lane 1: 100-bp ladder, lane 2: 4a-3A, lane 3: 4a-3B, lane 4: Ag55, lane 5: Sua.40 and lane 6: GeneRuler 1-kb Plus DNA Ladder. Expected band sizes are 492 bp for 2La and 207 bp for 2L + a (46). **Figure S2.** Passage number does not affect the molecular assay to differentiate cell lines. Molecular fingerprints for three PCRs amplifying indel regions in gDNA isolated from 4a-3A cells at passage 4 (lanes 2, 7, 12) and passage 29 (lanes 3, 8 and 13) and gDNA isolated from Sua4.0 cells at passage 5 (lanes 4, 9 and 14) and passage 57 (lanes 5, 10 and 15). Indel 2R.25670547 (lanes 2–5), indel 3R.11788474 (7–10), indel 3R.11809836 (lanes 12–15) and 1-kb Plus DNA Ladder (lanes 1, 6 and 11). Band sizes are as expected; for indel 2R.25670547 282 bp in 4a-3A and 361 in Sua4.0, for indel 3R.11788474 735 bp for 4a-3A cells and 736 bp for Sua4.0 cells with the single nucleotide difference not resolvable on an agarose gel or important for cell line authentication and for indel 3R.11809836 263 bp in 4a-3A cells and 262 bp in Sua4.0 cells. As 4a-3A cells originate from the 4a r/r strain of mosquitoes and Sua4.0 cells from the Suakoko 2La strain of mosquitoes, minor differences in sequence are expected [5].

## Data Availability

The sequencing data generated from 4a-3A and 4a-3B cell lines has been deposited in the SRA under accession number PRJNA842429.
