# Supplementary material for: Molecular characterization and genetic authentication assay for Anopheles ‘hemocyte-like’ cell lines 4a-3A and 4a-3B
Source: Parasit Vectors. 2022 Dec 13;15:465. doi: 10.1186/s13071-022-05590-3 (PMC9749150; doi:10.1186/s13071-022-05590-3)
Supplement: Supplementary file 1 — Additional file 1: Table S1. Primer sequences for indels selected for cell line diagnostic. Fasta file of indel region sequences. Sequence of the three diagnostic indel regions, 2R_25670547, 3R_11788474 and 3R_11809836, in each of the four tested cell lines. 2La inversion. Figure S1. Molecular karyotyping of the 2La inversion in four cell lines. Sua4.0 cells are fixed for the 2La inversion, and 4a-3A, 4a-3B and Ag55 cells are fixed for 2L + a. Lane 1: 100-bp ladder, lane 2: 4a-3A, lane 3: 4a-3B, lane 4: Ag55, lane 5: Sua.40 and lane 6: GeneRuler 1-kb Plus DNA Ladder. Expected band sizes are 492 bp for 2La and 207 bp for 2L + a (46). Figure S2. Passage number does not affect the molecular assay to differentiate cell lines. Molecular fingerprints for three PCRs amplifying indel regions in gDNA isolated from 4a-3A cells at passage 4 (lanes 2, 7, 12) and passage 29 (lanes 3, 8 and 13) and gDNA isolated from Sua4.0 cells at passage 5 (lanes 4, 9 and 14) and passage 57 (lanes 5, 10 and 15). Indel 2R.25670547 (lanes 2–5), indel 3R.11788474 (7–10), indel 3R.11809836 (lanes 12–15) and 1-kb Plus DNA Ladder (lanes 1, 6 and 11). Band sizes are as expected; for indel 2R.25670547 282 bp in 4a-3A and 361 in Sua4.0, for indel 3R.11788474 735 bp for 4a-3A cells and 736 bp for Sua4.0 cells with the single nucleotide difference not resolvable on an agarose gel or important for cell line authentication and for indel 3R.11809836 263 bp in 4a-3A cells and 262 bp in Sua4.0 cells. As 4a-3A cells originate from the 4a r/r strain of mosquitoes and Sua4.0 cells from the Suakoko 2La strain of mosquitoes, minor differences in sequence are expected [5]. [file 13071_2022_5590_MOESM1_ESM.pdf]

>4a3A_2R_25670547
GAATGCAAATATGGTTTGCTCTAAGTCACACAAATGGTGGTGCAAAGCCAATGACGCATTTCCAAAACCTGGT
ATTAAC
TAAGTACAGTCAACTCCCTCTTATCCGCAATCCAATGGGACTGGCGGAGTGAAGTAGAGGTTTGCACATTGCA
GAGATT
GAAATGTTCTTACGAGCTTTAAGAACATTGAAGAATCAATAATGTCCTGAAAACGTTGCTTGAACAGTTGCGA
CAGAAA
GCTTGGCGTCCTTGGATG
>4a3B_2R_25670547
GAATGCAAATATGGTTTGCTCTAAGTCACACAAATGGTGGTGCAAAGCCAATGACGCATTTCCAAAACCTGGT
ATTAAC
TAAGTACAGTCAACTCCCTCTTATACGCAAACCAATGGGACTGGCGGAGTGAAGTAGAGGTTTGCACATTGCA
GAGATT
GAAATGTTCTTACGAGCTTTAAGAACATTGAAGGATCAATAATGTGTTGGAATAGTCGCTTGAACAGTTGCGA
CAGAAA
GCTTGGCTTGCAGACATAATTCGAACAAGTTTTGTAATCTTTTATTTTTTGCATCCAAGGCCAGAAGAGAAGA
TACAAC
AAAACGTGGCCTTGGATG
>Sua4.0_2R_25670547
GAATGCAAATATGGTTTGCTCTAAGTCACACAAATGGTGGTGCAAAGCCAATGACGCATTTCCAAAACCTTGT
ATTAAC
TAAGTACAGTCAACTCCCTCTTATCCGCAATCCAATGGGACTGGCGGAGTGAAGTAGAGGTTTGCACATTGCA
GAGATT
GAAATGTTCTTACGAGCTTTAAGAACATTGAAGAATCAATAATGTGCTGAAAACGTTGCTTGAACATATGCTA
CAGAAA
GCTTGGCTTGCAGACATAATTCTAACAAGTTTTGTAACCTTTTATTTTTTGCATCCAAGGCCAGAAGAGAAGA
TACAAC
AAAACGTGTCCTTGGATG
>Ag55_2R_25670547
GAATGCAAATATGGTTTGCTCTAAGTCACACAAATGGTGGTGCAAAGCCAATGACGCATTTCCAAAGCCTGGT
ATTAAC
TAAGTACAGTCAACTCCCTCTTATCCGCAATCCAATGGGACTGGCGGAGTGAAGTAGAGGTTTGCACATTGCA
GAGATT
GAAATGTTCTTACGAGCTTTAAGAACATTGAAGAATCAATAATGTGCTGAAAACGTTGCTTGAACATATGCTA
CAGAAA
GCTTGGCTTGCAGACATAATTCTAACAAGTTTTGTAATCTTTTATTTTTTGCATCCAAGGCCAGAAGAGAAGA
TACAAC
AAAACGTGTCCTTGGATG
>4a3A_3R_11788474
GAGCTTGGTATGTTTACGAACTAGTTTGAATTTAATGCCAGTTTATCAGAAATGTTTGTGGATTCCTGACACA
AATTAT
AATCGCGATTTGCCATTGAGGGGATACTATTCCATGTATTGTAGATGATATTACTGATCTTGTGGAGATGTAG
GTTACA
ATTTTATATAGTCCATATGGCGAGGATTTGCAGATTGATAAACTTATTAATATATACTTTAAAAGCAAAGAAA
TGTTGA
ACAACAATGTTAAGCGTTTCGTTTGTTTTTCATAGAACTATCCTCAATTTTCACCCTTTATTGTCACTACAGA
ATAAAA
ATCGATTGTTTGTATACCGCTTCATTGAAGCTAATCTGGTTGAATTTCTGAAATACTGCTATTCCAATTTCGA
AAAAAG
CTTTCAAGTCCATTGCATATACGCAGATTTTCATGCAATTTCAGGGTGGTCCTGTGGTACAGTCGTCAACTCG
AACGTC
TCAATAACACGCCCGTAATAGGTTCAAGCCCGGAATGGACCGTCCCCTCGTAGTAAGGATTTTGACTCCCGGC
TACGTG

GTATTGAATAAAGTCTTGAGGGCCTGTATAGGCCGGCATGTCCGCGTAGGACGTTACGCCAAATAGAAGAAGA
TTTTCA
TGCAGCTTTTGATAAATTCAACCATATCTTACTTCTAGCGAAACTGATAAAATATGAAGTAAGCAGAGA
>4a3B_3R_11788474
GAGCTTGACATGTTTACGAACTAGTTAGAATTCAATGCCAGTTTATCAGATATATTTGTGGATTCCTGGCACA
AATTAT
AATCGCTATTTGCCATTGAGGGGATACTATTCCATGTATTGTAGATGATATTACTGATCTTGTGGAGATGTAG
GTTACA
ATTTTCTGTAGTCCATATGGCGTATACCGCTTCATTGAAGCTATTCTGGTTAAATGTCTGAAATACTGCTATT
CCAAAT
TCGATAAAAGTTTTCAAGTCAATTGCATATACGCAGATTTTCATGCAGCTTTTGATAAATTCAACCATATCTT
ACTTCT
AGCGAAACTGATAAAATATGAAATAAGCAAAGA
>Sua4.0_3R_11788474
GAGCTTGGCATGTTTACGAACTAGTTTGAATTTAATGCCAGTTTATCAGAAATGTTTGTGGATTCCTGGTACA
AATTTA
ATCGCTATTTGCCACTGAGGGGATACTATTCCATGTATTGTAGATGATATTACTGATCTTGTTGAGATATAGG
TTACAA
TTTGCTGTAGTCCATATGGCGAGGATTTGCAGATTGATAAACTTATTAATATATACTTTGAAAGAAAAGAAAT
ACCGAA
CAGCGATGTTAAGCGTTTCGTTTGTTTTTCGTAGAACTATCCTCAATGTTCACCCTTTATTGTCACTACAGAA
TAATCA
TCGATTGTTTGTATACCGCTTCATTGAAGCTAATCGGGTTGAATTTCTAAAATACTGCTATTCCAATTTCGAT
AAAAGC
TTTCAAGTCCATTGCATATGCGCAGATTTTCATTCAATTTCAGGGTGGTCCTGTGGTACAGTCGTCAACTCGA
ATGTCT
CAATAACACGCCCGTAATAGGTTCAAGCCCGGAATGGACCGTCCCCTCGTAGTAAGGATTCTGACTCCCGGCT
ACGTGG
TATTGAATAAAGTCTTGAAAGCCTGTATAGGCCGGCATGTCCGCGTAGGACGTTACGCCAAATAAAAGAAGAT
TTTCAT
GCAGCTTTTGATAAATTCAACCATATCTTACTTCTAGCGAAACTGATAAAATATGAAGTAAGCAAAGA
>Ag55_Allele1_3R_11788474
GAGCTTGGCATGTCTACGAACTAGTTTGAATTTATTGCCAGTTCATGAGATATATTTGTGGATTCCTAGCACA
AAATAT
AATCAAATTATAAAATTTATAACAAAATATAATGTATTGTAGATGATATTACTGATCTTGTGGAGATGTAGGT
TACAAT
TTTCTGTAGTCCATATGGCGTATACCGCTTCATTGAAGCTAATCTGGTTGAATTTCTGAAACACTGCTATTCC
AATTTC
GATAAAGGTTTTCAAGTCAATTGCATATACGCATATTTTCATGCAGCTTTTGATAAATTCAACCATATCTTAC
TTGTAG
CGAAACTGATAAAATATGAAGTAAGCAAAGA
>Ag55_Allele2_3R_11788474
GAGCTTGACATGTTTACGAACTAGTTTGAATTCAATGCCAGTATATCAGATATATTTGTGGATTCCTGGCACA
AATTAT
AATCGCTATTTGCCATTGAGGGGATACTATTCCATGTATTGTAGATGATATTACTGATCTTGTGGAGATGTAG
GTTACA
ATTTGCTGTAGTCCATATGGCGTATACCGCTTCATTGAAGCTATTCTGGTTAAATGTCTGAAATACTGCTATT
CCAAAT
TCGATAAAAGTTTTCAAGTCAATTGCATATACGCAGATTTTCATGCAGCTTTTGATAAATTCAACCATATCTT
ACTTCT
AGCGAAACTGATAAAATATGAAGTAAGCAAAGA
>4a3A_3R_11809836
AACATAATGTGGGCGAGCGAGGATCGTGTTCGAATGAGTGTGTTTATTTCCCTTGTTGCTGTCTGCTATCTGT
GTTGGG

CTACCATAATTATGTCCCTTTGGACAGTCCACTGCTACAGCACGAGCACCCTGGATATGCCATTAATGTAATT
TATCCC
GTTGGGCTGTCGATGAGTTGAATACAAAGAGCCGACAGGTGGATAGGTTTAGATAAGAAAGAAAAACGAAGTA
AGTCTG
T
>4a3B_3R_11809836
AACATAATGTGGGCGAGCGAGGATCGTGTTCGAATGAGTGTGTTTATTTCCCTTGTTGCTGTCTGCTATCTGT
GTTGGG
CTACCATAATTATGTCCCTTTGGACAGTCCACTACTACAGCACGTAAGGCTGT
>Sua4.0_3R_11809836
AACATAATGTGGGCGAGCGAGGATCGTGTTCGAATGAGTGTGTTTATTTCCCTTGTTGCTGTCTGCTATCTGT
GTTGGG
CTACCATAATTATGTCCCTTTGGACAGTCCACTGCTACAGCACGAGCACCCTGGATATACCATTAATGTAATT
TATCCC
GTTGGGCTGTCGATGAGTTGAATACAAAGAGCGACAGGTGGATAGGTTTAGATAAGAAAGAAAAACTACGTAA
GGCTGT
>Ag55_Allele1_3R_11809836
AACATAATGTGGGCGAGCGAGGATCGTGTTCAAATGAGTGTGTTTATTTCCCTTGTTGCTGTCTGCTATCTGT
GTTGGG
CTACCATAATTATGTCCCTTTGGACGGTCCACTACTACAGCACGAGCACCCTGGATATGCCATTAATGTAATT
TATCCC
GTTGGGATGAGTTGAATACAAAGAGCGACAGGTGGATAGGCTTAGATAAGAAAGAAAAACGACGTAAGGCTGT
>Ag55_Allele2__3R_11809836
AACATAATGTGGGCTAGCGAGGATCGTGTTCGAATGAGTGTGTTTATTTCCCTTGTTGCTGTCTGCTATCTGT
GTTGGG
CTACCATAATTATGTCCCTTTGGACAGTCCACTACTACAGCACGAGCACCCTGGATATGCCATTAATGTAATT
TATCCC
GTTGGGCTGTCGATGAGTTGAATACAAAGAGCGACAGGTGGGATGGGTTTAGATAAGAAAGAAAAACGACGTA
AGGCTG
T

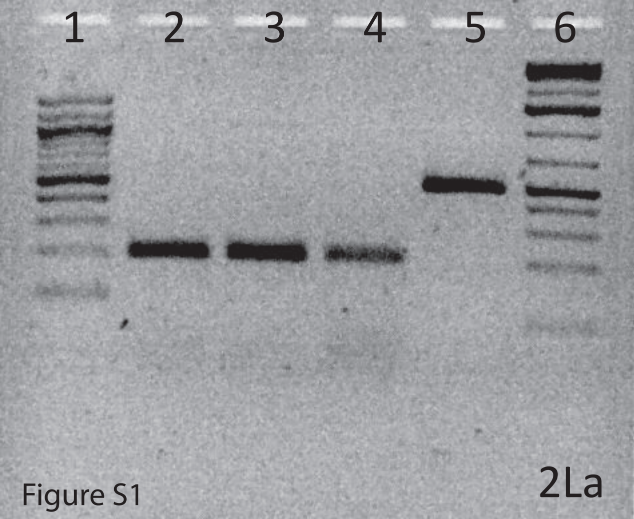
1
2
3
4
5
6
Figure S1
2La

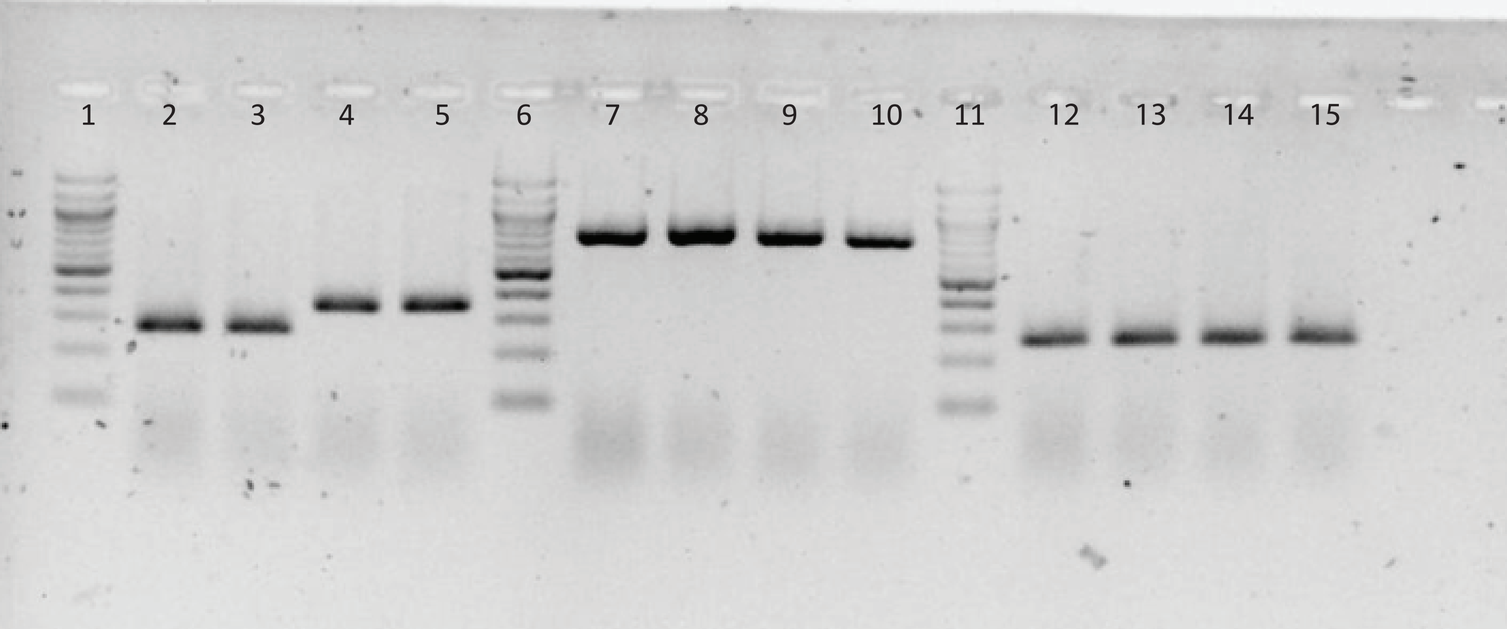

Figure S2

Table S1. Primer sequences for indels selected for cell line diagnostic

**Name**	**Sequence (5’-3’)**
2R.25670547_F	TGAATCACCGTGGAATGCAA
2R.25670547_R	TGCTTATTGTCAGACCATCCAAG
3L.40012707_F	AAACGGCAAGCGGGAATGAT
3L.40012707_R	GGATAAAGCTWGGAACGCCC
3R.11632056_F	TGACATTCTTCGTGTGCTCC
3R.11632056_R	CGTACGCCGTGACATGTATG
3R.11788474_F	CTTTGTCTACTTACTGGGAGCT
3R.11788474_R	TGAAGCCAATATACGAGGTCT
3R.11809836_F	CCGCAACGCCGTAACATAATG
3R.11809836_4a3A_R	AGCCATATACTACACAGACTTAC
3R.11809836_4a3B_R	AGCAATATACTACACAGCCTTAC
